# Exploiting the Bayesian approach to derive counts of married women of reproductive age across Cameroon for healthcare planning, 2000–2030

**DOI:** 10.1038/s41598-022-23089-w

**Published:** 2022-10-27

**Authors:** Raïssa Shiyghan Nsashiyi, Md Mizanur Rahman, Lawrence Monah Ndam, Masahiro Hashizume

**Affiliations:** 1 Department of Global Health Policy, Graduate School of Medicine, The University of Tokyo, Tokyo, Japan; 2 Institute for Nature, Health, and Agricultural Research (INHAR), Yaoundé, Cameroon; 3 Hitotsubashi Institute for Advanced Study, University of Hitotsubashi, 2-1 Naka, Kunitachi, Tokyo, 186-8601 Japan; 4 Department of Agronomic and Applied Molecular Sciences, Faculty of Agriculture and Veterinary Medicine, University of Buea, Buea, Cameroon

**Keywords:** Public health, Health policy

## Abstract

Estimates of married women of reproductive age (MWRA) are needed for policy decisions to enhance reproductive health. Given the unavailability in Cameroon, this study aimed to derive MWRA counts by regions and divisions from 2000 to 2030. Data included 1976, 1987, and 2005 censuses with 606,542 women, five Demographic and Health Surveys from 1991 to 2018 with 48,981 women, and United Nations World Population Prospects from 1976 to 2030. Bayesian models were used in estimating fertility rates, net-migration, and finally, MWRA counts. The total MWRA population in Cameroon was estimated to increase from 2,260,665 (2,198,569–2,352,934) to 6,124,480 (5,862,854–6,482,921), reflecting a 5.7 (5.2–6.2) percentage points (%p) annual rise from 2000–2030. The *Centre* and *Far North* regions host the largest numbers, projected to reach 1,264,514 (1,099,373–1,470,021) and 1,069,814 (985,315–1,185,523), respectively, in 2030. The highest divisional-level increases are expected in *Mfoundi* [14.6%p (11.2–18.8)] and *Bénoué* [14.9%p (11.1–20.09). This study’s findings, showing varied regional- and divisional-level estimates of and trends in MWRA counts should set a baseline for determining the demand for programmes such as family planning, and the scaling of relevant resources sub-nationally.

## Introduction

Married women of reproductive age (MWRA), i.e., women aged 15–49 years who are married or in a union, form a population of key interest in reproductive, maternal, newborn, child, and adolescent health. They make up the base population for evaluating progress in important Sustainable Development Goal (SDG 3.7^1^) indicators related to reproductive health, such as fertility^2^, contraceptive use, unmet need, and demand satisfied for family planning^3,4^. Estimates and projections of MWRA counts are thus necessary for monitoring these indicators, and for planning interventions relevant to enhancing reproductive health. Thus far, MWRA counts produced by the United Nations Population Division have enabled reproductive health monitoring at the country-level^3^. Still, given increasing recognition of substantial within-country disparities in reproductive health progress^4,5^, subnational estimates of MWRA counts have become necessary for tracking trends and inequalities in relevant indicators, and for making informed policy and decisions about the allocation of reproductive health resources, particularly in resource-limited settings.

For many developing nations, the lack of accurate population registers and unavailability or poor quality of population data warrants for subnational population figures on MWRA to be constructed using alternative data sources. Common data sources include surveys and censuses, and ‘administrative’ data such as tax and electoral records^6^. These, however, tend to substantially vary by country and contain varied depths of information that usually determine whether or not they can be used for subnational population estimation^6,7^. Importantly, data should provide information on key demographic indicators such as mortality or survival, fertility, and migration, whose assumptions are crucial for determining the future course of populations^8^. Considering the limitations in data, demographers have developed varied population estimation methods that range from simple trend interpolation and extrapolation to model-based approaches that depend on the relationships between selected demographic determinants and changes in a population over time^9^.

Increasingly, Bayesian methods in demography have become useful in subnational population estimation as they provide a framework for integrating data from diverse sources into model settings that allow for information exchange across country regions and time while accounting for uncertainty in data^7,10^. These features are important given substantial variabilities that exist in demographic trends across sub-regions within a country^6,11^ and increased data uncertainty for smaller geographical areas and population sizes at the subnational scale^7^.

Employing the Bayesian methods to derive subnational estimates of MWRA populations would be useful in a setting such as Cameroon which lacks recent, comprehensive, and reliable population data. Recent assessments have placed Cameroon among countries with slow progress in terms of key reproductive health outcomes^3^ and shown increasing within-country disparities in wealth-related progress towards the met demand for family planning^12^. Subnational counts of MWRA are thus necessary for more comprehensive assessments of geospatial disparities in reproductive health performance. And should also facilitate the distribution of reproductive health resources, including, infrastructure, personnel, and funding, according to the base population size per health administrative area. This study adopts a Bayesian approach for quantifying subnational populations to estimate counts of married women of reproductive age by first- (*Regions*) and second-level (*Divisions*) administrative units of Cameroon from 2000 to 2030.

## Methods

### Setting

Cameroon is a lower-middle-income country located at latitudes 2º and 13º North and longitude 9º and 16º East, between West and Central Africa. It has a surface area of about 475,400 km^2^ and a population estimated at 24.6 million in 2021^13^. The country is partitioned into 10 first-level, 58 s-level, and 360 third-level administrative units, named regions, divisions, and sub-divisions, respectively^13^. Its health sector is mostly organized in line with the administrative units into; three levels of health administration, i.e., central (ministry), intermediary (regional delegations), and peripheral (health districts) levels; and three levels of health care delivery including, tertiary, secondary, primary health facilities^14^. Health resource allocations are estimated at 63.6 US$ for annual per capita health budget; and health facility and Human resources for Health (HRH) densities at 1 per 5000 and 1.7 per 1000 inhabitants, respectively^14,15^.

### Data sources

Three sources of data were used, including; census data for Cameroon conducted in 1976, 1987, and 2005, made available through Integrated Public Use Microdata Series (IPUMS) International^16^; Demographic and Health Survey (DHS) data for Cameroon for 1991^17^, 1998^18^, 2004^19^, 2011^20^, and 2018^21^; and the 2019 World Population Prospects (WPP) life tables for Cameroon^22^. The data summary is included in the Supplementary (Table S1).

### Variables

The outcome variable of population counts of MWRA by single age, for married women 15–49 years, and by division and year was extracted from census data. Net-migration, fertility, and survival components were incorporated as predictors.

### Net-migration

An internal net-migration component that was derived by single age, for married women 15–49 years, division, and year using two census data variables (GEO2_CM and MIGCM1). GEO2_CM indicates the division of current residence while MIGCM1 represents the third-level administrative unit (Sub-division) of previous residence. MIGCM1 sub-divisions were regrouped under corresponding divisions to derive a variable for the previous division of residence. Net-migration was then computed as the difference between the sum of MWRA in a division ‘a’ who indicated that they resided in a different previous division (in-migrants), and the sum of MWRA in other divisions who indicated a division ‘a’ as their previous division of residence (out-migrants). Population and net-migration counts were person-weighted because these are 10% sample microdata of the population. Net-migration counts for the age groups within divisions were verified by cross-checking that their sum equals the total for corresponding regions. The net migration component by age was computed as a proportion of total net migration (i.e., the net-migration in age group a/total net-migration per annum). Due to changes in administrative boundaries, population and net-migration counts for 12 and 4 divisions as per the 1976 and 1987 censuses, respectively, were further proportionately partitioned to match the current 58 divisions as in the 2005 census. Net-migration proportions for MWRA were estimated using a Bayesian model organized within a hierarchical framework of single age within divisions, divisions within regions, and in a time series approach to capture trends over time (Supplementary, page 4).

### Fertility

Age-specific fertility rates (ASFR) were computed from DHS data using the Stata software module ‘tfr2’^23^. ASFR were derived by single age, for women 15–49 years of women of reproductive age (WRA), by region and year. These were weighted by an all-women factor to reflect fertility rates for WRA in the entire population. A Bayesian hierarchical model (BHM) was then used to estimate ASFR at the regional levels (Supplementary, pages 3–4).

### Survival

WPP life tables for the years 1976 to 2030, national-level age-specific survival probabilities by single age, for women 15–49 years were derived. Via linear interpolation and extrapolation (‘ipolate’ in Stata) the five-year probabilities of surviving between the ages *x* and *x* + *5*, *5px*, were converted to single-age and single-year probabilities of surviving between the ages *x* and *x* + *1*, *1px*.

### Statistical analyses

Statistical analysis to estimate the population of MWRA by single age 15–49 years, for each division and region of Cameroon, builds on standard regression forecasting approaches that assume factors influencing population size to have measured effects on population change over time^9,24^. In this multiple regression approach, we expand upon these with reference to proposed Bayesian methodologies on small-area population forecasting^6,7^ incorporating as covariates, age-specific vital rates for fertility and survival, and net-migration proportion, which are demographic drivers that are considered to be of key interest to the dynamics of populations of females^25^. Age-specific estimates derived from the fertility and net-migration models as well as survival probabilities interpolation were used.

The model for population estimation was constructed^6,26^ such that for each age of MWRA, changes in the log-transformed population size of MWRA will be influenced by patterns of age-specific fertility and survival rates, and net-migration proportion for the corresponding ages within divisions and regions, and built in a time series approach to captures trends over time. The expected number of women aged* a* in division* d* within region *r* at time *t* was generated based on the general relation modelled on the log-scale based on assumptions of a normal distribution as;

1 $${\mathrm{log}\eta }_{adrt} \sim N\left({\mathrm{log}\eta }_{adrt }^{*}+{\mathrm{log\upepsilon }}_{adrt} \right)$$

2 $$\mathrm{log}{\eta }_{adrt }^{*}={\beta }_{0,adrt}+{\beta }_{1,adrt}.{X}_{1,adrt}+{\beta }_{2,adrt}.{X}_{2,adrt}.{X}_{3,adrt}+{u}_{1,adt} +{u}_{2,drt}+{\varepsilon }_{adrt}$$

where; ${\eta }_{adrt }^{*}$ equals the expected number of MWRA age *a* in division *d* region *r* at time *t*; ${\beta }_{0,adrt}$ is the random intercept term; ${\beta }_{1,adrt}$ and ${X}_{1,adrt}$ are the coefficient and observation associated with the net-migration proportion for MWRA aged *a* in region *r* at time *t*; ${\beta }_{2,a,d,r,t}$ shows the coefficient for the interaction term fertility and survival, ${X}_{2,adrt}$ and ${X}_{3,adrt}$ are the observations associated with fertility and survival for WRA aged *a* in division *d* region *r* at time *t*; ${u}_{1,adt}$ is the random slope defined by age *a* and division *d* at time *t*, and ${u}_{2,drt}$ is the random slope set per division d and region *r* at time *t*; and ${\varepsilon }_{adrt}$ is the residual of the hierarchical model. Other model specifications are included in the Supplementary (page 4).

Standard regression forecasting model proceeds in two steps. In step one, the relationship between the dependent variable and the independent variables is established by estimating the $\beta$ vectors^27^. This model established a set of relationships between changes in the population (dependent variable) with changes in the demographic drivers (independent variables) for each corresponding age, division, and region over time. In step one, time $(t)$ corresponds with the census years $t$, $t+11$, and $t+29$ representing observations for 1976, 1987, and 2005, respectively. In step two, out-of-sample population predictions for years from 2000 to 2030 that represent the periods between $t+24$ to $t+54$ were derived based on the relationships established in step one. These predictions incorporate values at $t+24$ to $t+54$ for each of the demographic drivers as estimated from their respective models. An important assumption for this approach is to establish that the independent variables are approximately linearly related to the dependent variable (Supplementary Table S2) and also not highly correlated^27^, hence the interaction term fertility and survival with r = 0.62 (Supplementary Table S3).

### Computations

Best estimates for all parameters of interest for each model were computed as the median of generated samples from the posterior distributions via a Markov Chain Monte Carlo (MCMC) algorithm. Final estimates of MWRA counts for each administrative unit and year are the corresponding aggregates of the median of posterior samples for the ages 15–49 years. The percentage change in the population over time was calculated as the median of the annual rates of change derived from the posterior samples. All 95% Credible Intervals equal the 2.5th and 97.5th percentiles of the samples. The sample size of the posterior distribution was set at 5000 MCMC for the fertility model and 10,000 MCMC for the net-migration and population models, each with the burn-in at 3000 MCMC following a check for effective sample size via ‘bayesstats ess’ command in Stata. For each model standard diagnostics were conducted to evaluate the convergence of MCMC including visual observations of trace plots for when the outputs of chains overlapped^28^, and quantitatively, the Gelman-Rubin diagnostics for values close to 1 and at most < 1.1^28^. Additionally, the posterior predictive p-values (PPPs) for the replicated outcomes and simulated residuals were checked to determine the agreement between replicated and observed data. PPPs are presented in Supplementary Table S4. PPPs < 0.05 and > 0.95 indicate model misfit^29^. The sensitivity of results was examined through two model validation exercises, including, the exclusion of predictors, and altering priors for the hyperparameters (Supplementary, page 5). Statistical analysis was conducted using Stata Statistical Software: Release 17.1.

## Results

Overall, the MWRA population in Cameroon is estimated at 2,260,665 [95% Credible Interval (CrI) 2,198,569–2,352,934] in 2000, and projected to reach 6,124,480 (5,862,854–6,482,921) (Table 1). The estimated counts and annual rates of change of the MWRA populations for each region for years between 2000 and 2030, are presented in Table 1.

**Table 1:** Married women of reproductive age counts and annual rates of change by regions of Cameroon and year.

Region	Population estimates (95% credible interval)				Annual rate of change in percentage points (95% credible interval)	
	2000	2010	2020	2030	2000–2030	2020–2030
Adamawa	137,484 (126,887–150,722)	184,659 (170,249–203,078)	252,072 (230,898–278,659)	348,829 (316,935–389,232)	5.12 (4.18–6.19)	3.83 (2.29–5.65)
Centre	337,354 (308,837–372,379)	504,928 (453,153–567,398)	786,405 (694,948–897,812)	1,264,514 (1,099,373–1,470,021)	9.15 (7.39–11.23)	6.08 (3.75–8.76)
East	109,582 (101,355–119,104)	134,833 (124,644–146,971)	167,296 (154,202–182,861)	208,402 (191,192–228,854)	3.01 (2.38–3.72)	2.48 (1.21–3.85)
Far north	495,044 (459,370–544,047)	633,579 (586,315–697,416)	818,946 (752,899–903,269)	1,069,814 (985,315–1,185,523)	3.86 (3.13–4.73)	3.06 (1.66–4.60)
Littoral	283,893 (252,532–323,984)	400,442 (351,774–460,816)	576,482 (500,737–668,230)	841,799 (719,875–990,906)	6.54 (4.95–8.41)	4.61 (2.26–7.42)
North	272,069 (245,418–307,632)	419,150 (374,787–476,919)	662,131 (584,961–763,594)	1,066,891 (939,031–1,237,530)	9.70 (7.86–12.01)	6.10 (3.63–8.97)
Northwest	164,941 (155,251–177,073)	206,518 (193,574–222,891)	265,193 (247,290–287,444)	350,104 (324,120–382,673)	3.73 (3.10–4.42)	3.20 (1.98–4.53)
West	227,774 (213,797–244,309)	266,688 (249,286–287,714)	321,025 (298,745–347,603)	396,079 (366,033–432,364)	2.47 (1.95–3.03)	2.34 (1.25–3.58)
South	74,957 (69,615–81,124)	110,865 (102,836–120,326)	167,628 (155,156–182,221)	258,435 (238,728–281,302)	8.16 (7.06–9.39)	5.40 (3.92–7.00)
Southwest	157,567 (146,614–170,596)	194,928 (181,005–212,463)	247,275 (227,822–270,308)	319,614 (293,832–351,933)	3.42 (2.76–4.18)	2.93 (1.63–4.40)
National total	2,260,665 (2,198,569–2,352,934)	3,056,589 (2,959,792–3,192,383)	4,264,453 (4,105,920–4,477,013)	6,124,480 (5,862,854–6,482,921)	5.69 (5.23–6.20)	4.36 (3.61–5.18)

### Regional-level MWRA population estimates

**Figure 1:**
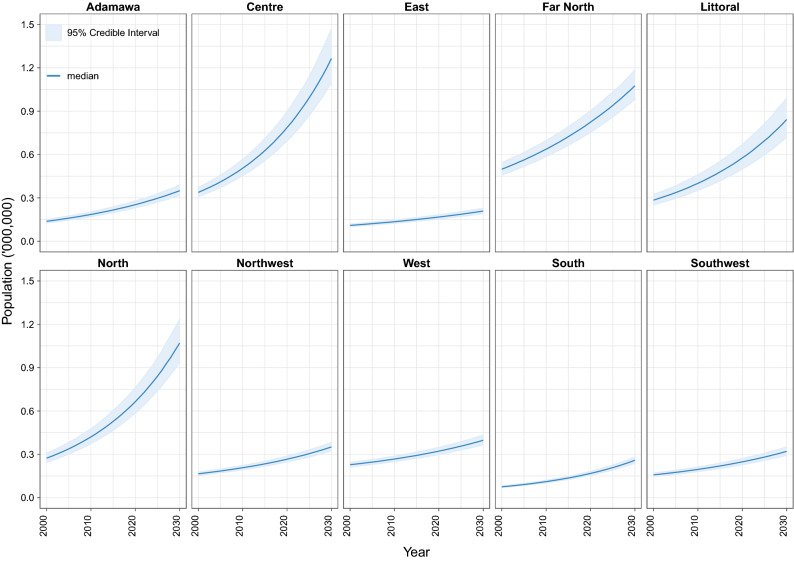
Estimated populations of married women of reproductive age by regions of Cameroon, 2000–2030.

Figure 1 shows the MWRA population levels and trends by regions of Cameroon from 2000 to 2030. The largest populations were estimated in the *Centre* and *Far North* regions, where the number of MWRA are projected to reach 1,264,514 (1,099,373–1,470,021) and 1,069,814 (985,315–1,185,523), respectively, in 2030 (Table 1). By these estimates, approximately 38.1% (35.6–41.0%) of the total population of MWRA will be concentrated in these two regions in 2030. Closely following these are the MWRA populations of the *North* and *Littoral* regions that were each estimated to surpass 800 thousand women in 2030. The smallest estimates were observed in the *East* and *South* regions, where 208,402 (191,192–228,854) and 258,435 (238,728–281,302) of MWRA are projected to reside in 2030.

### Divisional-level MWRA population estimates

**Figure 2:**
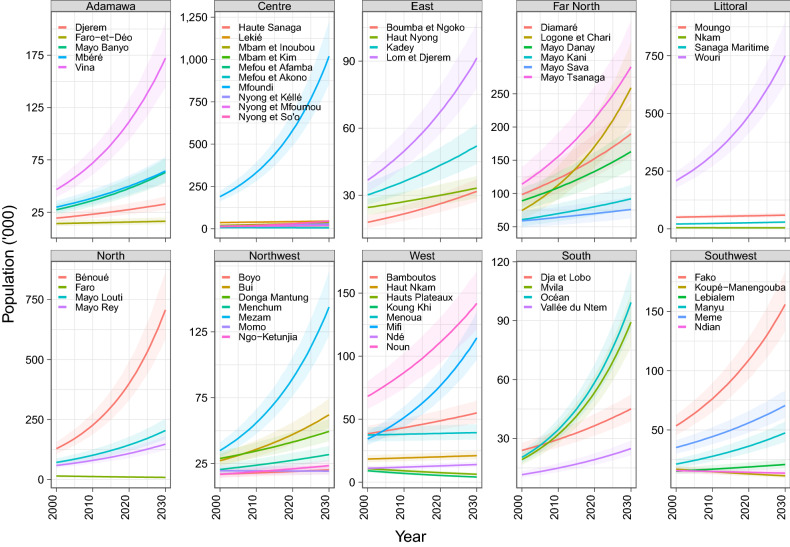
Married women of reproductive age population estimates by divisions within regions of Cameroon, 2000–2030. Lines and accompanying shaded areas represent the median and 95% credible intervals, respectively.

Figure 2 presents the MWRA population trends across the different divisions within each of the regions, from 2000 to 2030. The steepest annual rates of increase in the population of MWRA over this period were registered in the *Bénoué* division, 14.99 percentage points (%p) (11.11–20.09), within the *North* region, and the *Mfoundi* division within the Centre region 14.57%p (11.17–18.77) (Fig. 4). Three divisions with the highest counts of MWRA include *Mfoundi* in the Centre, *Wouri* in the *Littoral*, and *Bénoué* in the *North* regions. With 2030 projections of 1,019,032 (853,313–1,223,262), 749,306 (627,411–897,205), and 706,967 (586,917–866,890), their quarter of MWRA will represent 80.6% (77.6–83.2), 89.0% (87.2–90.5, and 66.3% (62.5–70.1) of their respective regional totals. Most other divisions show relatively moderate or slow increases in the MWRA populations, with median projections at ≤ 300 thousand in 2030. Declining trends were registered across several divisions, most steeply in *Koung Khi* [−1.83%p (−2.09 to −1.51)] and *Hauts Plateaux* [−1.39%p (−1.73 to −0.98)] in the *West*, and *Faro* [−1.34%p (−1.78 to −0.81)] in the *North*, between 2000 and 2030. The smallest divisional-level MWRA projections for 2030 were estimated at 4117 (3533–4834) for *Hauts Plateaux* in the *West*, 4399 (3777–5145) for *Nkam*, in the *Littoral*, and 5712 (4907–6663) for *Mefou et Akono* in the *Centre*. Supplementary Table S5 includes MWRA counts and annual rates of change by divisions of Cameroon.

**Figure 3:**
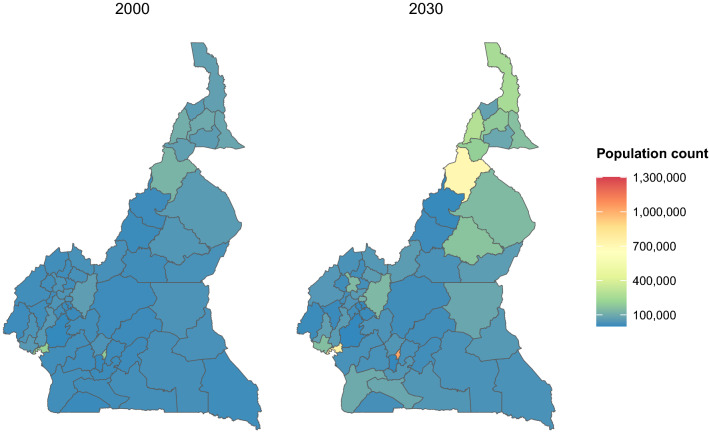
Geospatial distribution of married women of reproductive age populations by divisions of Cameroon: 2000 and 2030. Population = median married women of reproductive age estimates. Maps were generated using R programming environment, version 4.1.3 (https://cran.r-project.org/bin/windows/base/old/4.1.3/).

**Figure 4:**
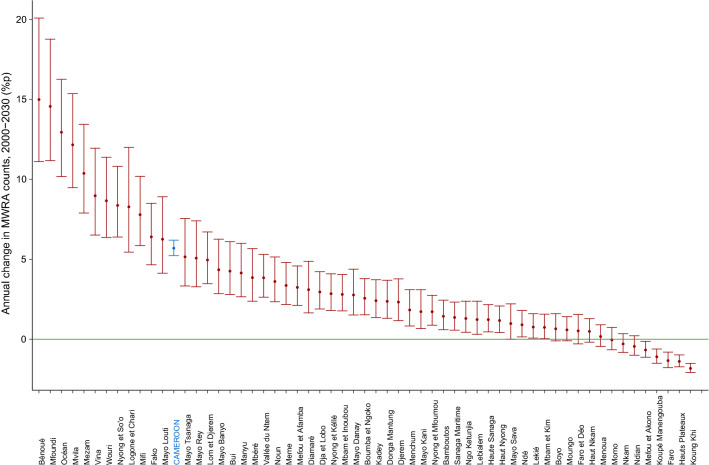
Annual changes in the married women of reproductive age populations by divisions of Cameroon. *MWRA*  married women of reproductive age, points = median change; horizontal lines = 95% credible intervals; blue plot = country-level; green line = mark for zero.

In Fig. 3, the geospatial distribution of the population shows higher concentrations of MWRA in the northern divisions of the country, where median projections for more than half divisions exceed 160,000 each in 2030. The rates of change in MWRA counts by divisions between 2000 and 2030 are ordered in Fig. 4. In 45 out of the 58 divisions, MWRA populations show median (including 95% CrI) changes of > 0%p annually.

### Sensitivity analysis

Excluding predictors (net-migration proportion, ASFR, and survival probability) for the MWRA population projections, the median absolute (and %) differences between the two sets of results were minor: 1577 (0.07%), 1666 (0.05%), 1346 (0.03%), and 133 (0.00%) overall, for 2000, 2010, 2020, and 2030, respectively. Also, the differences derived after altering priors for the hyperparameters were also small. The outputs are presented in Supplementary Table S6.

## Discussion

This is the first study to estimate subnational populations of MWRA for all the ten regions and 58 divisions of Cameroon, over the period 2000–2030. At the minimum, the population of MWRA is estimated to more than double over the 30-year study period. The results also suggest varied annual MWRA population growth across all regions and in at least three-quarters of the 57 divisions.

At the level of the regions, the steepest upward trends are seen in the *North*, *Centre*, *Littoral*, and *Far North*, which besides recording the highest numbers of MWRA, are also the four most populated regions of Cameroon^13^. Particularly accelerated increases were also seen in the most MWRA populated divisions, i.e., *Mfoundi*, *Bénoué*, and *Wouri*. These estimates are not unexpected as these three divisions recorded notably higher positive levels of net-migration (i.e., higher in-migration). In the case of the *Mfoundi* and *Wouri*, which are respectively the administrative and economic capitals, the pull factor for in-migration is likely due to accessibility to jobs and social amenities. *Mfoundi* serves as the seat of government that harbours administrative services at the highest level while *Wouri* is the hub of the industrial region and economic capital of Cameroon. Interestingly, a similar pattern of in-migration-driven growth in the WRA population has been shown across counties in Kenya^7^. With regards to the overall higher concentration of the MWRA population in the northern divisions, this could be related to the relatively higher fertility and polygamy rates, and lower median age at first marriage (16 years) in these Muslim-dominated parts of Cameroon^30,31^. Although currently unavailable, data on marriage rates at the subnational levels could have further uncovered reasons for some of the observed differences in the MWRA population trends.

The *East* and *South* with the smallest numbers of MWRA align with their fractions of the overall national population, as these two are the least-populated regions in the country^13^. Such is also the case at the second-level administrative units as *Mefou et Akono* and *Nkam* are the least-populated divisions^13^. Notably, most of these divisions that likewise rank bottom within their respective regions in terms of MWRA counts, i.e., *Mefou et Akono*, *Koupé Manengouba*, *Faro*, *Hauts Plateaux*, and *Koung Khi*, showed exclusive (including 95% CrI) negative annual rates of change. Trends should, however, be interpreted with caution as estimates do not indicate negative populations of MWRA, but rather decreasing numbers. Because estimates here are primarily time-based, positive or negative trends would be dependent on patterns in the raw data, in which case we only had three population data points between 1976 and 2005. Meanwhile, estimates especially for recent years require further consideration given the five-year-long ongoing civil conflict in the country that has resulted in displacements of populations in some parts of the *Northwest* and *Southwest* regions^32^. Therefore, future censuses will be crucial to capture trends in the post-conflict period and will most likely show smaller numbers in the aforementioned regions.

Of importance, subnational estimates of MWRA counts set a baseline for understanding the approximate demand for or actual numbers of women in need of family planning services per administrative unit. Numbers of women with or without access to related services, i.e., the supply and unmet demand for family planning, can also be determined via translations of indicator proportions into numbers. Estimates of MWRA populations have previously been used to determine the numbers of women with access to contraceptives, unmet need, and demand for family planning in Kenya^33^, India^4^, and Nigeria^5^. Such information should facilitate the establishment of quantitatively informed policies and programmes relevant to enhancing reproductive health. Meanwhile, MWRA counts could also serve for the measurement of other important indicators like fertility rates and maternal mortality in this base population^7^.

This study’s findings on regional- and divisional-level estimates of and trends in the MWRA populations are also crucial as they could inform the allocation and long-term scaling of reproductive health resources sub-nationally. For instance, in 2030, approximately two-thirds to three-quarters of available reproductive health resources should be allocated to 4 out of the 10 regions that are projected to host around 18.8–22.7% (*Centre*), 16.8–18.3% (*Far North*), 16.0–19.1% (*North*), and 12.3–15.3% (*Littoral*) of the national total of MWRA. Also, allocations for the *Mfoundi*, *Bénoué*, and *Wouri* divisions should be expanded as per the annual rate of increase in the MWRA population of around 4.4–11.3, 3.9–12.4, and 2.5–8.7%p, respectively, between 2020 and 2030. These details are important as the current distribution of health resources, including facilities, personnel, and budget, is uneven with the demand for healthcare in terms of population size^15,34^. The *Far North* which makes up 18% of the total population, for example, receives less than a third of the annual per capita health budget (≈1US$), less than half of health facilities (8.2%), and less than half of Human resources for Health (0.9 per 1000) allocated to the similarly populated *Centre* region (19.0%)^13–15^. Within the context of Cameroon, the study findings should inform the subnational allocation of resources such as, “Mother and child health Care/Family planning” centres, family planning providers, obstetrician-gynaecologist doctors and nurses, and contraceptive services and commodities including recently introduced modern contraceptives DMPA-SC injectable, Implanon NXT, and Sayana Press^35,36^.

Other advantages of the study have to do with the estimation approach that integrates within a formal Bayesian framework modelled projections of crucial demographic processes to derive probabilistic projections of MWRA populations at every age between 15 and 49 years. Importantly, the generated estimates are mostly similar to patterns of national-level MWRA populations for Cameroon that has been produced by the United Nations (Supplementary, Fig. S1). With slight adjustments, the methods should be applicable for estimating different sub-populations of interest sub-nationally across a range of less developed countries with similar data. Effects of demographic components on population forecasts are otherwise assumed to be fixed relations with deterministic methods or negligible with methods such as interpolation and extrapolation^7^. Increasingly, probabilistic projections of demographic drivers that form the basis for country-level population projections by the United Nations^37^ are being adopted for subnational forecasting^6,7^. Applying such probabilistic approaches at the subnational scale minimizes the effects of information volatility on demographic trends^6^. These are crucial because differences in population estimates can have large effects on measures of health indicators^7^.

Notwithstanding the realisations of this study, there are important limitations. First, unmarried WRA were excluded, even though, they make up an increasing share of users of family planning services^38^. Still, separate estimations are preferable given the difficultly to define the demand or need for family planning in the unmarried population^38,39^. Also, regional-level fertility and national level survival data were used to estimate populations at the level of divisions. However, the covariates take on different values within each subnational unit depending on the level of hierarchy within the Bayesian model^26^. While there could be trade-offs with the use of WRA survival probabilities, the WRA fertility rates would more likely reflect on future populations of MWRA in general^6,8^. Furthermore, due to data limitations, the census question on ‘previous residence’ was assumed to capture long-term internal migration as opposed to specific time-period migration as computed elsewhere^7^. In capturing migration patterns, another limitation exists as the values rather show the net-difference in internal migration rather than the actual numbers of people moving in and out of a location per period, given the likelihood for external-in and out-migration. In this regard, the net-migration proportion was adopted to incorporate patterns in the relative differences instead. Lastly, general uncertainties that arise in population forecasting due to the indirect impacts of non-demographic social and economic processes were not taken into consideration. Although these are harder to predict, they are often also linked to demographic processes^40^ that were factored in this study.

## Conclusions

This study presents varied estimates of and trends in populations of MWRA for the various regions and divisions of Cameroon that are based on a Bayesian hierarchical model built on components of the population and migration counts and fertility and survival statistics. In 2030, Cameroon’s overall population of MWRA is projected to be at least two times the estimated total in 2000. Annual growth patterns are seen in all regions and 45 of the 58 divisions. Of the total of MWRA in 2030, at least two-fifth and one-third are to reside in only three regions (*Centre, Far North,* and *North*) and three divisions (*Mfoundi Wouri*, and *Bénoué*), respectively. Importantly, the MWRA counts should serve as grounds for determining the levels of demand, supply, and unmet demand for key reproductive health services such as family planning, and as a rationale for defining what quotas to allocate and at what rate to expand available resources for each subnational unit in Cameroon. Future work will investigate how measures of different reproductive health indicators vary by the regional- and divisional-level estimates of MWRA populations.

## Supplementary Information

Supplementary Information.

## Data Availability

Data generated and/or analysed during this study are openly available via institutions as cited; Integrated Public Use Microdata Series (IPUMS) International, https://international.ipums.org/international/; Demographic and Health Survey, https://dhsprogram.com/data/available-datasets.cfm: and 2019 World Population Prospects by the United Nations, https://population.un.org/wpp/Download/Standard/CSV/.
