# Supplementary material for: Exploiting the Bayesian approach to derive counts of married women of reproductive age across Cameroon for healthcare planning, 2000–2030
Source: Sci Rep. 2022 Oct 27;12:18075. doi: 10.1038/s41598-022-23089-w (PMC9613669; doi:10.1038/s41598-022-23089-w)
Supplement: Supplementary file 1 — Supplementary Information. [file 41598_2022_23089_MOESM1_ESM.pdf]

Supplemental appendix

Title: Exploiting the Bayesian approach to derive counts of married women of reproductive age across Cameroon for healthcare planning, 2000–2030

Authors information

Raïssa Shiyghan Nsashiyi[1,2*], Md Mizanur Rahman[3], Lawrence Monah Ndam[4], Masahiro Hashizume[1]

[1]Department of Global Health Policy, Graduate School of Medicine, The University of Tokyo, Japan
[2]Institute for Nature, Health, and Agricultural Research (INHAR), Yaoundé, Cameroon
[3]Hitotsubashi Institute for Advanced Study, University of Hitotsubashi, 2-1 Naka, Kunitachi Tokyo 186-8601, Japan
[4]Department of Agronomic and Applied Molecular Sciences, Faculty of Agriculture and Veterinary Medicine, University of Buea, Cameroon
[*] Corresponding author: raissa.nsashiyi@gmail.com

Table S1. Variables and data used to estimate married women of reproductive age counts sub-nationally in Cameroon

Variables	Data	Year	Sample size	Target population
**Population and Net-migration count**	IPUMS-International Census data	1976	146,973	Married Women aged 15–49 years
		1987	163,784	
		2005	295,785	
**Age-specific fertility rate**	Demographic and Health Survey (DHS)	1991	3,871	Women aged 15–49 years
		1998	5,501	
		2004	10,656	
		2011	15,426	
		2018	13,527	
**Age-specific survival probability**	WPP-life tables	1976 – 2030	NA	

IPUMS=Integrated Public Use Microdata Series; WPP=World Population Prospects. Sub-divisions (i.e., third-level administrative units) serve as the smallest geography for the collection of IPUMS data. DHS includes 24 households per cluster. Clusters are randomly sampled and meant to be representative of estimates per region (i.e., first-level administrative units) of Cameroon. NA=Not applicable

Table S2. Results of regression of components for population estimation analysis

Variable		Regression coefficient
**Dependent**	**Independent**	
**Log MWRA counts**	Survival probability	-1.3×10*
	Fertility rate	0·3×10*
	Net-migration	9.8×10^{2}*

MWRA=married women of reproductive age; *p < 0·001. Net-migration = net-migration proportion

Table S3. Pairwise correlation table of variables used for the population projection

			**Age-specific**		
		Log MWRA counts	Net-migration proportion	Fertility rate	Survival Probability
**Age-specific**	Log MWRA counts	1.0000			
	Net-migration	0.6982*	1.0000		
	Fertility rate	0.2863*	0.0718*	1.0000	
	Survival Probability	0.1203*	0.0943*	0.6177*	1.0000

*p < 0·05.

Table S4. Check for goodness-of-fit for fertility, net-migration, and population projection models

Model		T	Mean	Standard Deviation	E(T_obs)	P(T>=T_obs)
Age-specific	Fertility (ln)	Mean	-.0000784	0.0217568	0.000656	**0.49660**
		Variance	10.9745	0.3942873	10.95948	**0.50825**
	Net-migration proportion	Mean	0.0000000273	0.0000067	0.00000105	**0.4818**
		Variance	0.00000499	0.000000114	0.00000171	**0.4965**
	Population (ln)	Mean	0.00000459	0.001073	-0.0000421	**0.5087**
		Variance	0.1301038	0.0029628	0.1300692	**0.5052**

Note: T and T_obs denote the test statistics computed using the replicated data and observed data, respectively. P(T>=T_obs) shows the posterior predictive p-value (PPP). PPPs compare the means and variances of the simulated residuals with those of the observed residuals. PPPs close to 0 or 1 indicate a lack of fit and close to 0.5 indicate very good agreement between the simulated and the observed residuals. ln=log-transformed outcome variable.

Fertility model

A Bayesian hierarchical model (BHM) was used to estimate age-specific fertility rates (ASFR) for women of reproductive age (WRA) at the regional levels. The model was built to capture regularities in fertility patterns within the standardised age structure for the fertility cycle, i.e., 15–49 years[1]. Similar to the one-parameter approach for estimating changes in patterns of fertility[1,2], estimation was conducted in a time-series approach that assumes changes in fertility rates to be captured via overall patterns per single year of age with random effects defined for age groups within regions. The expected ASFR for women aged *a* in region *r* at time *t* was derived based on the general relation modelled on the log-scale as and assuming a normal distribution as;

$$\log f_{art} \sim N(\log f^{*}_{art} + \log \varepsilon_{art})$$
$$\log f^{*}_{art} = \beta_{0,art} + z_{1,art} + \varepsilon_{art}$$

Where; f^{*}_{adrt} equals the expected ASFR for WRA age a in region *r* at time *t*; $\beta_{0,art}$ is the random intercept term, $z_{1,art}$ is the random slope of the fertility observations for each age a and region *r* at time *t*; and ε_{art} the residual of the hierarchical model. Based on this prior established

relationship over time from t (i.e., 1991 DHS) to $t + 27$ (i.e., 2018 DHS), out-of-sample predictions of ASFR were derived by single age of WRA and region from 1976–2030.
Model priors were specified as: $\beta_{0,art} \sim N(0, 100)$; $z_{1,art} \sim N(\{\log f^*_{art}: \beta_0\}, \sigma^2_{1,art})$; $\sigma^2_{1,art} \sim igamma(0.01, 0.01)$

Net-migration model

Net-migration counts for MWRA were estimated using a Bayesian model organized within a hierarchical framework of single age within divisions, divisions within regions, and in a time series approach to capture trends over time. Here, historical levels of net-migration for each age schedule are assumed to be dependent on unknown parameters that are only time-dependent as proposed by Lee (1993)[1,3]. The expected net-migration for women aged *a* in division *d* within region *r* at time *t* was modelled based on the general relation and assuming a normal distribution as specified below;

$$\log m_{adrt} \sim N(\log m^*_{adrt} + \log \varepsilon_{adrt})$$

$$m^*_{adrt} = \beta_{0,adrt} + I_{1,adt} + I_{2,drt} + \varepsilon_{adrt}$$

Where; m^*_{adrt} equals the expected net-migration for MWRA age *a* in division *d* region *r* at time *t*; $\beta_{0,adrt}$ is the random intercept term; $I_{1,adt}$ is the random effects of the observations for each age *a*, in division *d* at time *t*; and $I_{2,drt}$ is the random effects of the observations for each division *d* in region *r* at time *t*; ε_{adrt} is the residual of the hierarchical model. Based on the above relationship for the available census years, out-of-sample predictions of net-migration counts were also derived by single age of MWRA (15–49 years) and division from 1976–2030. Priors for the net-migration model were specified as follows: $\beta_{0,adrt} \sim N(0, 100)$; $I_{1,adt} \sim N(0, \sigma^2_{1,adt})$; $I_2 \sim N(0, 100)$; $\sigma^2_{1,adt} \sim igamma(0.01, 0.01)$

Estimates for age-specific net-migration included negative values that indicate higher levels of out-migration.

Population projection model

Priors for the population model were specified as: $\beta_{0,adrt} \sim N(0, 100)$; $\beta_{1,adrt} \sim N(0, 100)$; $\beta_{2,adrt} \sim N(0, 100)$; $u_{1,adt} \sim N(0, \sigma^2_{1,adt})$; $u_{2,drt} \sim N(0, 100)$; $\sigma^2_{1,adt} \sim igamma(0.01, 0.01)$

Sensitivity analysis

Check for sensitivity of results for MWRA counts was examined via (1) the exclusion of covariates, and (2) altering priors for the hyperparameters.

(1) Assessing the role of covariates: As in previous studies[4], predictors (i.e., net-migration proportion, ASFR, and survival probability) were excluded from the model, and then a comparison was made between the median absolute and % differences in MWRA estimates. The differences were minor as reported in Table S6.

(2) Altering priors for the hyperparameters: Here, hyperparameters were assigned weakly informative instead of vaguely-informative prior (hyper-prior) distributions specified in the main model. Hyperparameters are shared by all intercept coefficients to borrow strength and facilitate parameters smoothing from each group[5,6]. Thus, in assigning hyperparameters, there are benefits of complete-pooling and no-pooling for the model.

In our main model, priors for β $(\beta_0, \beta_1, \beta_2)$ was assigned a normal distribution with mean = zero and standard deviation = 100, $\beta_0, \beta_1, \beta_2 \sim N(0, 100)$. For hyper-priors, both u_1 and u_2 were assigned normal distributions with mean = zero and standard deviations = σ_1^2 (with inverse gamma distribution) and100, respectively.

Gelman *et al*., (2008), advocate for proper but weak prior distribution, since separation is rarely an issue in the case of linear regression[7]. Nonetheless, we still check for collinearity since this is an issue that still could arise. For this sensitivity analysis, the standard deviation for u_1 was replaced 0. Thus, specifying $N(0, 100)$ hyperpriors for both u_1 and u_2. Median absolute and % differences in the posterior distributions are presented in Table S6.

Table S5. Married women of reproductive age counts and annual change by Cameroon's administrative units and year

REGION<br>Division	Population Estimates (95% Credible Interval)				Annual rate of change in percentage points (95% Credible Interval)	
	2000	2010	2020	2030	2000–2030	2020–2030
**ADAMAWA**	**137484 (126887–150722)**	**184659 (170249–203078)**	**252072 (230898–278659)**	**348829 (316935–389232)**	**5.12 (4.18–6.19)**	**3.83 (2.29–5.65)**
Djerem	19359 (16211–23368)	23114 (19321–27850)	27464 (22956–33388)	32797 (27348–39926)	2.32 (1.16–3.78)	1.93 (-0.47–5.04)
Faro et Déo	14175 (11749–17265)	14882 (12343–18122)	15607 (12943–19058)	16372 (13565–20045)	0.52 (-0.30–1.55)	0.49 (-1.75–3.36)
Mayo Banyo	27400 (23006–32825)	36154 (30276–43817)	47869 (40072–57749)	63107 (52956–76268)	4.34 (2.85–6.26)	3.21 (0.58–6.50)
Mbéré	29890 (25115–36036)	38528 (32442–46481)	49745 (41782–59974)	64391 (53636–77648)	3.85 (2.38–5.66)	2.94 (0.30–6.07)
Vina	46659 (39337–55767)	71981 (61039–86356)	111387 (94083–133168)	172161 (144505–206357)	8.97 (6.51–11.95)	5.42 (2.46–9.29)
**CENTRE**	**337354 (308837–372379)**	**504928 (453153–567398)**	**786405 (694948–897812)**	**1264514 (1099373–1470021)**	**9.15 (7.39–11.23)**	**6.08 (3.75–8.76)**
Haute Sanaga	13409 (11507–15592)	14909 (12842–17370)	16522 (14232–19276)	18326 (15741–21395)	1.22 (0.46–2.16)	1.10 (-0.80–3.35)
Lekié	36625 (31552–42666)	39309 (33714–45792)	42042 (36094–48963)	45079 (38699–52636)	0.76 (0.07–1.59)	0.72 (-1.14–2.90)
Mbam et Inoubou	20087 (17314–23407)	24631 (21195–28676)	30167 (25941–35150)	36999 (31782–43309)	2.80 (1.77–4.05)	2.26 (0.17–4.74)
Mbam et Kim	15468 (13362–17987)	16507 (14211–19201)	17680 (15159–20604)	18891 (16239–22081)	0.74 (0.03–1.57)	0.71 (-1.13–2.88)
Mefou et Afamba	13158 (11342–15281)	16493 (14199–19240)	20682 (17897–24163)	25947 (22276–30189)	3.24 (2.12–4.58)	2.53 (0.41–5.09)
Mefou et Akono	7161 (6175–8358)	6643 (5713–7753)	6163 (5292–7203)	5712 (4907–6663)	-0.68 (-1.12– -0.13)	-0.74 (-2.32–1.20)
Mfoundi	189628 (161356–222770)	332327 (280761–393958)	582203 (489853–692697)	1019032 (853313–1223262)	14.57 (11.17–18.77)	7.51 (4.36–11.31)
Nyong et Kéllé	15628 (13432–18222)	19215 (16496–22487)	23523 (20206–27401)	28942 (24856–33913)	2.85 (1.80–4.08)	2.31 (0.19–4.92)
Nyong et Mfoumou	13233 (11429–15369)	15218 (13133–17705)	17460 (15051–20351)	20042 (17275–23356)	1.72 (0.88–2.75)	1.47 (-0.45–3.85)
Nyong et So'o	12958 (11149–15070)	19676 (16880–22958)	29963 (25634 35074)	45544 (38845 53436)	8.37 (6.39–10.82)	5.19 (2.58–8.40)
**EAST**	**109582 (101355–119104)**	**134833 (124644–146971)**	**167296 (154202–182861)**	**208402 (191192–228854)**	**3.01 (2.38–3.72)**	**2.48 (1.21–3.85)**
Boumba et Ngoko	17963 (15426–21052)	21716 (18603–25478)	26285 (22475–30706)	31731 (27224–37277)	2.56 (1.53–3.79)	2.09 (-0.04–4.62)
Haut Nyong	24578 (21168–28512)	27153 (23422–31466)	30041 (25872–34928)	33202 (28650–38730)	1.17 (0.41–2.08)	1.05 (-0.78–3.23)
Kadey	30241 (25819–35604)	36231 (30818–42719)	43532 (36937–51386)	52081 (44011–62010)	2.41 (1.35–3.72)	1.98 (-0.20–4.66)
Lom et Djerem	36801 (31484–43092)	49733 (42641–58398)	67438 (57667–79096)	91387 (77788–107633)	4.96 (3.47–6.71)	3.56 (1.17–6.56)
**FAR NORTH**	**495044 (459370–544047)**	**633579 (586315–697416)**	**818946 (752899–903269)**	**1069814 (985315–1185523)**	**3.86 (3.13–4.73)**	**3.06 (1.66–4.60)**
Diamaré	98336 (80875–122233)	122694 (100954–152190)	152493 (125367–188960)	189773 (155235–235085)	3.10 (1.64–4.87)	2.45 (-0.37–5.95)
Logone et Chari	74364 (60391–94124)	112778 (91416–141436)	170887 (139195–215605)	258926 (210509–328820)	8.28 (5.45–12.00)	5.15 (1.53–9.85)
Mayo Danay	88908 (74271–108443)	108689 (90782–132437)	133096 (110372–162406)	162871 (135424–197775)	2.77 (1.51–4.38)	2.23 (-0.27–5.48)
Mayo Kani	60648 (50539–73890)	69632 (57862–84628)	80046 (66292–97959)	91908 (75945–112685)	1.72 (0.66–3.09)	1.51 (-0.94–4.48)
Mayo Sava	58755 (48153–73081)	64131 (52594–79882)	69726 (57287–86652)	75892 (62439–94711)	0.98 (0.00–2.21)	0.90 (-1.58–4.09)
Mayo Tsanaga	114034 (94293–140143)	155655 (129399–191528)	212698 (176804–260689)	290445 (240946–358395)	5.15 (3.33–7.55)	3.68 (0.72–7.46)
**LITTORAL**	**283893 (252532–323984)**	**400442 (351774–460816)**	**576482 (500737–668230)**	**841799 (719875–990906)**	**6.54 (4.95–8.41)**	**4.61 (2.26–7.42)**
Moungo	50344 (43347–58657)	53170 (45631–61951)	56073 (48081–65651)	59082 (50488–69704)	0.58 (-0.10–1.41)	0.54 (-1.25–2.69)
Nkam	4832 (4141–5647)	4682 (4014–5495)	4530 (3896–5300)	4399 (3777–5145)	-0.30 (-0.82–0.34)	-0.29 (-1.98–1.75)
Sanaga Maritime	20636 (17774–23932)	23105 (19945–27058)	25919 (22365–30147)	29012 (25017–33826)	1.36 (0.56–2.32)	1.21 (-0.71–3.43)
Wouri	208080 (177572–246873)	319485 (270794–378068)	489960 (414037–581707)	749306 (627411–897205)	8.66 (6.37–11.39)	5.31 (2.52–8.69)

Continued on next page …

Continuation …

REGION<br>Division	Population Estimates (95% Credible Interval) 2000	2010	2020	2030	Annual rate of change in percentage points (95% Credible Interval) 2000–2030	2020–2030
NORTH	272069 (245418–307632)	419150 (374787–476919)	662131 (584961–763594)	1066891 (939031–1237530)	9.70 (7.86–12.01)	6.10 (3.63–8.97)
Bénoué	128328 (106602–157148)	226431 (188292–277211)	399837 (329942–492949)	706967 (586917–866890)	14.99 (11.11–20.09)	7.65 (3.91–12.41)
Faro	14383 (11952–17676)	12111 (9996–14850)	10237 (8474–12565)	8604 (7149–10567)	-1.34 (-1.78– -0.81)	-1.60 (-3.39–0.74)
Mayo Louti	71086 (58434–88255)	100971 (83391–125191)	143789 (117958–178532)	204137 (168253–253074)	6.25 (4.13–8.91)	4.21 (1.04–8.21)
Mayo Rey	58272 (48208–71153)	79637 (66098–97541)	108268 (89431–132976)	147183 (122004–181060)	5.07 (3.28–7.40)	3.58 (0.74–7.17)
NORTHWEST	164941 (155251–177073)	206518 (193574–222891)	265193 (247290–287444)	350104 (324120–382673)	3.73 (3.10–4.42)	3.20 (1.98–4.53)
Boyo	16905 (14272–20148)	17935 (15209–21400)	19064 (16097–22780)	20214 (17024–24131)	0.65 (-0.11–1.59)	0.62 (-1.41–3.08)
Bui	27246 (23054–32493)	35872 (30360–42885)	47133 (39786–56284)	62019 (52402–74448)	4.26 (2.80–6.10)	3.15 (0.60–6.34)
Donga Mantung	28890 (24632–34300)	34561 (29339–41101)	41408 (35065–49188)	49414 (42039–59044)	2.37 (1.30–3.69)	1.93 (-0.33–4.70)
Menchum	20522 (17340–24716)	23791 (20025–28604)	27539 (23171–33221)	31836 (26662–38396)	1.83 (0.82–3.10)	1.56 (-0.70–4.38)
Mezam	34898 (29805–41250)	56008 (47587–66408)	89637 (76314–105974)	143800 (121922–170784)	10.37 (7.89–13.44)	6.03 (3.07–9.50)
Momo	19569 (16556–23359)	19487 (16560–23224)	19399 (16364–23184)	19318 (16351–23119)	-0.05 (-0.66–0.74)	-0.03 (-1.95–2.35)
Ngo Ketunjia	16911 (14356–20036)	18863 (16002–22380)	21013 (17923–24992)	23502 (19938–27975)	1.30 (0.43–2.38)	1.17 (-0.94–3.67)
WEST	227774 (213797–244309)	266688 (249286–287714)	321025 (298745–347603)	396079 (366033–432364)	2.47 (1.95–3.03)	2.34 (1.25–3.58)
Bamboutos	38455 (33015–44794)	43229 (37094–50343)	48717 (41679–57089)	54966 (46921–64808)	1.43 (0.59–2.44)	1.28 (-0.67–3.54)
Haut Nkam	18387 (15796–21544)	19229 (16521–22455)	20117 (17317–23500)	21095 (18062–24621)	0.49 (-0.19–1.29)	0.48 (-1.35–2.67)
Hauts Plateaux	10802 (9281–12624)	9034 (7776–10554)	7544 (6475–8839)	6305 (5372–7389)	-1.39 (-1.73– -0.98)	-1.64 (-3.10–0.11)
Koung Khi	9114 (7815–10654)	6983 (5990–8219)	5368 (4610–6301)	4117 (3533–4834)	-1.83 (-2.09– -1.51)	-2.32 (-3.69– -0.68)
Menoua	37448 (32164–43454)	38002 (32764–44306)	38663 (33341–45253)	39279 (33682–45936)	0.17 (-0.45–0.90)	0.16 (-1.58–2.31)
Mifi	34363 (29429–40068)	51278 (43824–60105)	76582 (65438–89946)	114472 (97224–135307)	7.79 (5.86–10.19)	4.94 (2.42–8.10)
Ndé	11035 (9474–12905)	11960 (10241–13961)	12938 (11131–15178)	13993 (12037–16419)	0.90 (0.14–1.80)	0.82 (-1.07–3.10)
Noun	68170 (58029–80214)	86973 (74085–102480)	111095 (94509–130786)	141852 (121070–167155)	3.61 (2.34–5.15)	2.78 (0.49–5.57)
SOUTH	74957 (69615–81124)	110865 (102836–120326)	167628 (155156–182221)	258435 (238728–281302)	8.16 (7.06–9.39)	5.40 (3.92–7.00)
Dja et Lobo	23890 (20615–27778)	29479 (25437–34385)	36454 (31346–42421)	45090 (38864–52334)	2.96 (1.89–4.22)	2.36 (0.24–4.85)
Mvila	19190 (16546–22334)	32038 (27628–37174)	53460 (46062–62231)	89233 (76853–104160)	12.16 (9.48–15.36)	6.70 (3.84–10.02)
Océan	20357 (17517–23600)	34471 (29764–40070)	58545 (50502–67750)	99288 (85525–115036)	12.95 (10.18–16.26)	6.95 (4.11–10.36)
Vallée du Ntem	11520 (9959–13369)	14877 (12845–17285)	19169 (16565–22349)	24824 (21322–28835)	3.84 (2.63–5.31)	2.95 (0.76–5.53)
SOUTHWEST	157567 (146614–170596)	194928 (181005–212463)	247275 (227822–270308)	319614 (293832–351933)	3.42 (2.76–4.18)	2.93 (1.63–4.40)
Fako	53474 (45747–62760)	76276 (65087–89842)	109136 (93315–127923)	155936 (133669–183723)	6.40 (4.66–8.50)	4.30 (1.83–7.39)
Koupé Manengouba	16834 (14433–19859)	14741 (12570–17277)	12885 (11053–15164)	11276 (9638–13265)	-1.10 (-1.50– -0.61)	-1.24 (-2.83–0.64)
Lebialem	15216 (12797–18319)	16886 (14241–20297)	18715 (15740–22501)	20820 (17544–25061)	1.23 (0.31–2.38)	1.12 (-1.06–3.89)
Manyu	21173 (17828–25463)	27740 (23388–33422)	36272 (30507–43776)	47453 (39854–57070)	4.14 (2.65–6.00)	3.08 (0.51–6.29)
Meme	35182 (30072–41309)	44350 (38179–52369)	56015 (47642–65935)	70600 (60200–82945)	3.36 (2.17–4.80)	2.62 (0.35–5.38)
Ndian	15688 (13279–18681)	14934 (12637–17805)	14253 (12075–16994)	13529 (11445–16177)	-0.45 (-1.00–0.21)	-0.48 (-2.32–1.70)
National Total	2260665 (2198569–2352934)	3056589 (2959792–3192383)	4264453 (4105920–4477013)	6124480 (5862854–6482921)	5.69 (5.23–6.20)	4.36 (3.61–5.18)

Table S6. Comparisons of posterior difference for main population projection model; vs model without predictors, and vs model with altered prior distribution on hyperparameters

**REGION** Division	**Panel A**				**Panel B**			
	**Posterior difference between the main model with vs the model without predictors, mean (%)**				**Posterior difference between the main model vs the model with altered hyper-priors, mean (%)**			
	**2000**	**2010**	**2020**	**2030**	**2000**	**2010**	**2020**	**2030**
**ADAMAWA**	**114 (0.08)**	**153 (0.08)**	**211 (0.08)**	**283 (0.08)**	**133 (0.10)**	**331 (0.18)**	**660 (0.26)**	**1211 (0.35)**
Djerem	18 (0.09)	24 (0.11)	32 (0.12)	43 (0.13)	13 (0.07)	29 (0.12)	49 (0.18)	77 (0.24)
Faro et Déo	13 (0.09)	15 (0.10)	18 (0.11)	20 (0.12)	2 (0.02)	4 (0.03)	5 (0.04)	10 (0.06)
Mayo Banyo	23 (0.08)	31 (0.09)	45 (0.09)	61 (0.10)	25 (0.09)	58 (0.16)	109 (0.23)	187 (0.30)
Mbéré	27 (0.09)	38 (0.10)	54 (0.11)	81 (0.13)	31 (0.10)	68 (0.18)	124 (0.25)	208 (0.32)
Vina	33 (0.07)	43 (0.06)	62 (0.06)	78 (0.05)	62 (0.13)	172 (0.24)	372 (0.33)	729 (0.42)
**CENTRE**	**190 (0.06)**	**96 (0.02)**	**-251 (-0.03)**	**-1139 (-0.09)**	**448 (0.13)**	**1093 (0.22)**	**2634 (0.33)**	**5243 (0.41)**
Haute Sanaga	13 (0.10)	17 (0.11)	21 (0.13)	26 (0.14)	10 (0.07)	14 (0.10)	25 (0.15)	29 (0.16)
Lekié	39 (0.11)	52 (0.13)	65 (0.15)	80 (0.18)	18 (0.05)	19 (0.05)	41 (0.10)	36 (0.08)
Mbam et Inoubou	22 (0.11)	33 (0.14)	48 (0.16)	69 (0.19)	17 (0.08)	30 (0.12)	60 (0.20)	79 (0.21)
Mbam et Kim	12 (0.08)	12 (0.07)	13 (0.08)	14 (0.07)	5 (0.03)	3 (0.02)	9 (0.05)	3 (0.02)
Mefou et Afamba	10 (0.08)	12 (0.07)	15 (0.07)	18 (0.07)	10 (0.07)	16 (0.10)	34 (0.16)	44 (0.17)
Mefou et Akono	7 (0.09)	7 (0.11)	7 (0.12)	7 (0.13)	0 (0.00)	-3 (-0.05)	-3 (-0.06)	-7 (-0.12)
Mfoundi	54 (0.03)	-82 (-0.02)	-477 (-0.08)	-1426 (-0.14)	347 (0.18)	926 (0.28)	2283 (0.39)	4765 (0.47)
Nyong et Kéllé	14 (0.09)	18 (0.10)	24 (0.10)	32 (0.11)	14 (0.09)	25 (0.13)	48 (0.20)	66 (0.23)
Nyong et Mfoumou	11 (0.08)	14 (0.09)	16 (0.09)	19 (0.10)	9 (0.07)	13 (0.09)	26 (0.15)	30 (0.15)
Nyong et So'o	9 (0.07)	12 (0.06)	16 (0.05)	23 (0.05)	20 (0.16)	50 (0.25)	112 (0.37)	199 (0.44)
**EAST**	**99 (0.09)**	**130 (0.10)**	**173 (0.10)**	**223 (0.11)**	**125 (0.11)**	**185 (0.14)**	**275 (0.16)**	**453 (0.22)**
Boumba et Ngoko	19 (0.11)	30 (0.14)	43 (0.16)	59 (0.19)	22 (0.12)	32 (0.15)	47 (0.18)	73 (0.23)
Haut Nyong	26 (0.10)	34 (0.13)	44 (0.15)	55 (0.16)	25 (0.10)	30 (0.11)	35 (0.12)	51 (0.15)
Kadey	24 (0.08)	27 (0.08)	32 (0.07)	36 (0.07)	30 (0.10)	39 (0.11)	52 (0.12)	80 (0.15)
Lom et Djerem	30 (0.08)	39 (0.08)	53 (0.08)	73 (0.08)	48 (0.13)	84 (0.17)	142 (0.21)	250 (0.27)
**FAR NORTH**	**386 (0.08)**	**504 (0.08)**	**643 (0.08)**	**829 (0.08)**	**336 (0.07)**	**728 (0.11)**	**1375 (0.17)**	**2285 (0.21)**
Diamaré	59 (0.06)	57 (0.05)	52 (0.03)	36 (0.02)	48 (0.05)	93 (0.08)	167 (0.11)	251 (0.13)
Logone et Chari	53 (0.07)	78 (0.07)	104 (0.06)	154 (0.06)	88 (0.12)	229 (0.20)	491 (0.29)	916 (0.35)
Mayo Danay	83 (0.09)	115 (0.11)	162 (0.12)	219 (0.13)	62 (0.07)	117 (0.11)	204 (0.15)	304 (0.19)
Mayo Kani	38 (0.06)	35 (0.05)	27 (0.03)	17 (0.02)	23 (0.04)	32 (0.05)	48 (0.06)	64 (0.07)
Mayo Sava	65 (0.11)	90 (0.14)	116 (0.17)	146 (0.19)	24 (0.04)	38 (0.06)	50 (0.07)	59 (0.08)
Mayo Tsanaga	88 (0.08)	128 (0.08)	183 (0.09)	257 (0.09)	90 (0.08)	219 (0.14)	415 (0.20)	693 (0.24)
**LITTORAL**	**43 (0.02)**	**-228 (-0.06)**	**-845 (-0.15)**	**-2128 (-0.25)**	**308 (0.11)**	**614 (0.15)**	**1201 (0.21)**	**2307 (0.27)**
Moungo	44 (0.09)	50 (0.09)	57 (0.10)	64 (0.11)	12 (0.02)	-3 (0.00)	17 (0.03)	-6 (-0.01)
Nkam	4 (0.09)	5 (0.10)	5 (0.10)	5 (0.11)	0 (0.01)	-2 (-0.03)	-1 (-0.02)	-3 (-0.07)
Sanaga Maritime	16 (0.08)	18 (0.08)	20 (0.08)	22 (0.08)	9 (0.04)	7 (0.03)	21 (0.08)	18 (0.06)
Wouri	-21 (-0.01)	-301 (-0.09)	-928 (-0.19)	-2219 (-0.30)	286 (0.14)	611 (0.19)	1164 (0.24)	2298 (0.31)

Continued on next page …

Continuation …

**REGION** Division	**Panel A**				**Panel B**			
	**Posterior difference between the main model with vs the model without predictors, mean (%)**				**Posterior difference between the main model vs the model with altered hyper-priors, mean (%)**			
	**2000**	**2010**	**2020**	**2030**	**2000**	**2010**	**2020**	**2030**
**NORTH**	**221 (0.08)**	**353 (0.08)**	**581 (0.09)**	**1005 (0.09)**	**391 (0.14)**	**970 (0.23)**	**2283 (0.34)**	**4808 (0.45)**
Bénoué	109 (0.08)	201 (0.09)	370 (0.09)	715 (0.10)	250 (0.20)	697 (0.31)	1703 (0.43)	3734 (0.53)
Faro	11 (0.08)	9 (0.08)	8 (0.08)	7 (0.08)	-7 (-0.05)	-23 (-0.19)	-28 (-0.27)	-32 (-0.37)
Mayo Louti	59 (0.08)	84 (0.08)	125 (0.09)	180 (0.09)	92 (0.13)	188 (0.19)	390 (0.27)	718 (0.35)
Mayo Rey	42 (0.07)	59 (0.07)	78 (0.07)	102 (0.07)	56 (0.10)	107 (0.13)	218 (0.20)	388 (0.26)
**NORTHWEST**	**136 (0.08)**	**172 (0.08)**	**209 (0.08)**	**252 (0.07)**	**134 (0.08)**	**282 (0.14)**	**578 (0.22)**	**950 (0.27)**
Boyo	17 (0.10)	22 (0.12)	27 (0.14)	33 (0.16)	7 (0.04)	8 (0.05)	14 (0.07)	13 (0.06)
Bui	22 (0.08)	29 (0.08)	39 (0.08)	52 (0.08)	28 (0.10)	61 (0.17)	115 (0.24)	176 (0.28)
Donga Mantung	23 (0.08)	27 (0.08)	32 (0.08)	35 (0.07)	22 (0.08)	38 (0.11)	69 (0.17)	90 (0.18)
Menchum	17 (0.09)	23 (0.10)	29 (0.11)	37 (0.12)	13 (0.06)	23 (0.10)	39 (0.14)	49 (0.15)
Mezam	24 (0.07)	33 (0.06)	39 (0.04)	46 (0.03)	54 (0.16)	144 (0.26)	326 (0.36)	615 (0.43)
Momo	19 (0.10)	23 (0.12)	26 (0.13)	29 (0.15)	1 (0.00)	-6 (-0.03)	-8 (-0.04)	-18 (-0.09)
Ngo Ketunjia	13 (0.08)	16 (0.08)	17 (0.08)	21 (0.09)	9 (0.06)	13 (0.07)	23 (0.11)	25 (0.11)
**WEST**	**208 (0.09)**	**259 (0.10)**	**333 (0.10)**	**429 (0.11)**	**150 (0.07)**	**360 (0.14)**	**584 (0.18)**	**890 (0.22)**
Bamboutos	34 (0.09)	40 (0.09)	49 (0.10)	59 (0.11)	23 (0.06)	45 (0.10)	58 (0.12)	65 (0.12)
Haut Nkam	14 (0.08)	14 (0.07)	14 (0.07)	15 (0.07)	6 (0.03)	10 (0.05)	8 (0.04)	4 (0.02)
Hauts Plateaux	11 (0.11)	12 (0.13)	12 (0.16)	12 (0.18)	-4 (-0.04)	-8 (-0.09)	-13 (-0.17)	-19 (-0.30)
Koung Khi	9 (0.10)	8 (0.11)	7 (0.13)	6 (0.15)	-7 (-0.07)	-12 (-0.17)	-17 (-0.31)	-21 (-0.50)
Menoua	40 (0.11)	50 (0.13)	61 (0.16)	72 (0.18)	7 (0.02)	12 (0.03)	5 (0.01)	-10 (-0.03)
Mifi	28 (0.08)	40 (0.08)	60 (0.08)	89 (0.08)	53 (0.15)	139 (0.27)	273 (0.36)	477 (0.42)
Ndé	9 (0.09)	10 (0.09)	12 (0.09)	13 (0.09)	4 (0.04)	7 (0.06)	7 (0.06)	4 (0.03)
Noun	62 (0.09)	84 (0.10)	118 (0.11)	164 (0.12)	67 (0.10)	167 (0.19)	263 (0.24)	388 (0.27)
**SOUTH**	**61 (0.08)**	**90 (0.08)**	**133 (0.08)**	**199 (0.08)**	**97 (0.13)**	**277 (0.25)**	**590 (0.35)**	**1133 (0.44)**
Dja et Lobo	24 (0.10)	34 (0.12)	49 (0.13)	69 (0.15)	19 (0.08)	43 (0.15)	73 (0.20)	108 (0.24)
Mvila	14 (0.07)	22 (0.07)	32 (0.06)	49 (0.05)	32 (0.17)	97 (0.30)	219 (0.41)	439 (0.49)
Océan	16 (0.08)	26 (0.07)	43 (0.07)	71 (0.07)	36 (0.18)	113 (0.33)	257 (0.44)	521 (0.52)
Vallée du Ntem	8 (0.07)	9 (0.06)	9 (0.05)	10 (0.04)	10 (0.09)	24 (0.16)	41 (0.22)	65 (0.26)
**SOUTHWEST**	**119 (0.08)**	**138 (0.07)**	**160 (0.06)**	**180 (0.06)**	**144 (0.09)**	**290 (0.15)**	**514 (0.21)**	**886 (0.28)**
Fako	36 (0.07)	42 (0.05)	47 (0.04)	48 (0.03)	74 (0.14)	171 (0.22)	324 (0.30)	580 (0.37)
Koupé Manengouba	13 (0.08)	11 (0.08)	10 (0.07)	8 (0.08)	-2 (-0.01)	-10 (-0.07)	-18 (-0.14)	-23 (-0.20)
Lebialem	12 (0.08)	14 (0.08)	16 (0.09)	19 (0.09)	10 (0.06)	14 (0.08)	18 (0.10)	26 (0.12)
Manyu	19 (0.09)	29 (0.11)	43 (0.12)	61 (0.13)	23 (0.11)	47 (0.17)	82 (0.23)	134 (0.28)
Meme	24 (0.07)	25 (0.06)	26 (0.05)	24 (0.03)	36 (0.10)	68 (0.15)	111 (0.20)	176 (0.25)
Ndian	15 (0.09)	16 (0.11)	18 (0.13)	19 (0.14)	4 (0.02)	0 (0.00)	-4 (-0.03)	-7 (-0.05)
**National Total**	**1577 (0.07)**	**1666 (0.05)**	**1346 (0.03)**	**133 (0.00)**	**2264 (0.10)**	**2167 (0.13)**	**2696 (0.17)**	**3129 (0.20)**

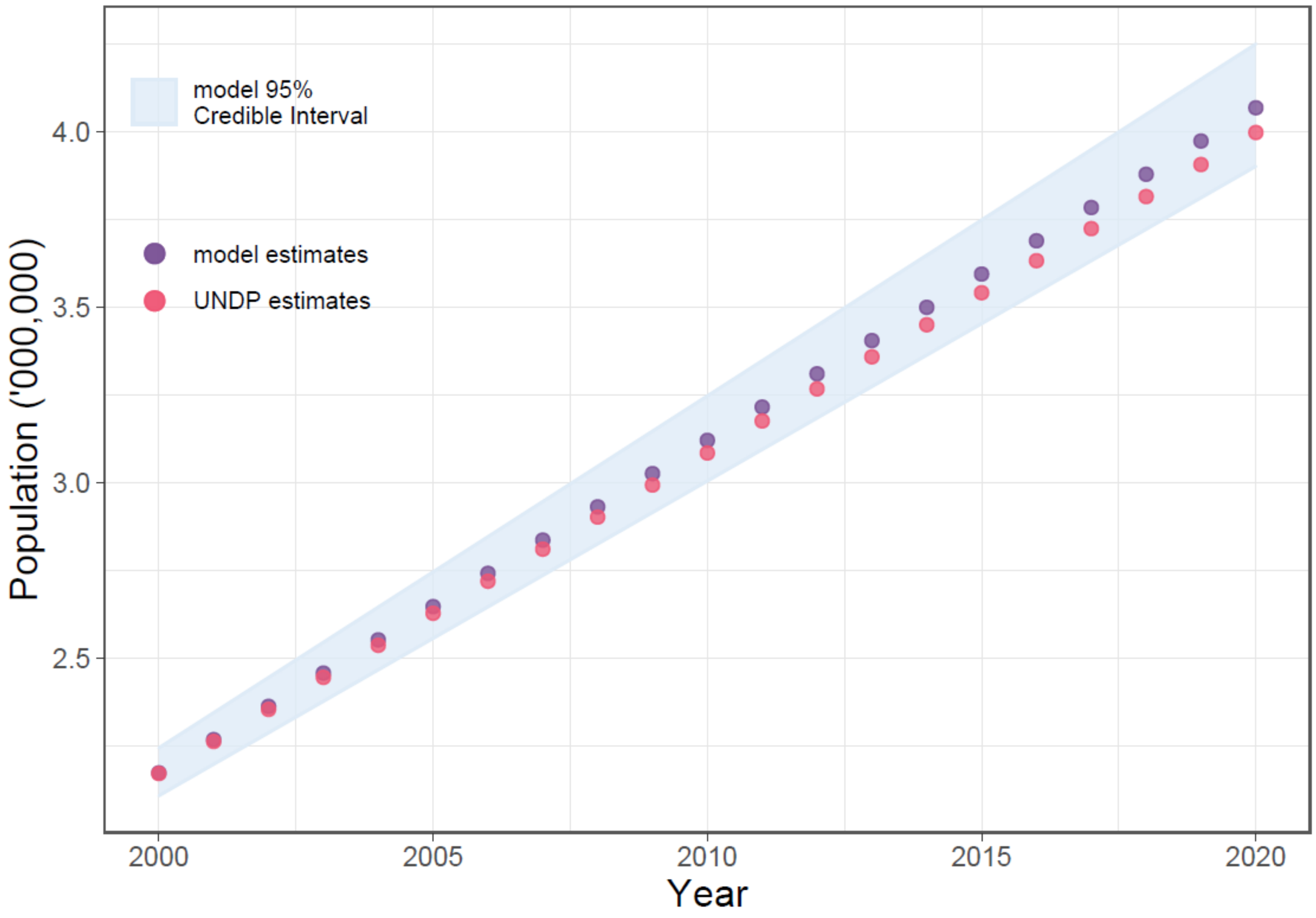

Figure S1: National total counts of married women of reproductive age in Cameroon, 2000–2020
Model estimates are those derived from this study. UNDP (United Nations Development Programme) estimates were downloaded from the UN's Family Planning Estimation Tool (FPET).

References

1. Lee, R.D. Modeling and forecasting the time series of US fertility: age distribution, range, and ultimate level. *International journal of forecasting* **9**, 187-202 (1993).
2. Thompson, P.A., Bell, W.R., Long, J.F. & Miller, R.B. Multivariate time series projections of parameterized age-specific fertility rates. *Journal of the American Statistical Association* **84**, 689-699 (1989).
3. García Guerrero, V. A Probabilistic Method to Forecast the International Migration of Mexico by Age and Sex. *Papeles de Población* **22**, 113-140 (2013).
4. Rahman, M.M.*, et al.* Trend and projection of skilled birth attendants and institutional delivery coverage for adolescents in 54 low- and middle-income countries, 2000-2030. *BMC medicine* **20**, 46 (2022).
5. Ntzoufras, I. *Bayesian modeling using WinBUGS*, (John Wiley & Sons, 2011).
6. Gelman, A. Prior distributions for variance parameters in hierarchical models (comment on article by Browne and Draper). *Bayesian analysis* **1**, 515-534 (2006).
7. Gelman, A., Jakulin, A., Pittau, M.G. & Su, Y.-S. A weakly informative default prior distribution for logistic and other regression models. *The annals of applied statistics* **2**, 1360-1383 (2008).
